# Under Pressure to Achieve? The Impact of Type and Style of Task Instructions on Student Cheating

**DOI:** 10.3389/fpsyg.2019.01624

**Published:** 2019-08-14

**Authors:** Caroline Julia Pulfrey, Maarten Vansteenkiste, Aikaterina Michou

**Affiliations:** ^1^Laboratory of Social Psychology, Institute of Psychology, Université de Lausanne, Lausanne, Switzerland; ^2^Department of Developmental, Personal and Social Psychology, Ghent University, Ghent, Belgium; ^3^Graduate School of Education, Bilkent University, Ankara, Turkey

**Keywords:** achievement goal complex, cheating, mastery-approach goals, autonomy-support, self-enhancement values

## Abstract

Combining principles of Achievement Goal Theory, which maintains that performance goals play a key role in individuals’ likelihood of cheating, and Self-Determination Theory, which highlights the importance of autonomy support and autonomous motivation underlying achievement goals, we examined whether the combination of experimentally inducing a mastery-approach (relative to performance-approach) goal with an autonomy-supportive manner (instead of controlling) may attenuate cheating. In two experiments carried out with university students, one classroom based (*N* = 164) and one laboratory (*N* = 160), we manipulated the type of induced goal (performance- vs. mastery-approach) and style of introducing the goal (i.e., controlling vs. autonomy-supportive) by taking also into consideration participants’ values. We hypothesized that the least behaviorally observed cheating would occur in a context promoting mastery-approach goals in an autonomy-supportive way and among individuals low in self-enhancement value adherence. The dependent variables in both studies consisted of two set of exercises, both including questions that could only be solved by cheating. Results of Poisson regression analyses revealed that in both studies the least cheating in the first set of exercises occurred in the autonomy-supportive/mastery-approach condition, indicating that this induced goal complex has the greatest potential to restrain academic dishonesty in the short-term. Interaction effects with self-enhancement value adherence revealed that the cheating inhibitory effects of this induced goal complex was less effective for those who value power and achievement.

## Introduction

Lance Armstrong stated, “it was impossible to win the Tour de France without doping” (Mandard, 2013). This anecdotic example suggests that Armstrong’s achievement goal of beating his opponents and the pressure he felt to achieve this goal combine to explain his unethical behavior. But do achievement goals and reasons for goal adoption, in isolation or in combination, contribute to dishonest behavior?

In this research, we address this question experimentally, integrating two well-established motivational frameworks, namely achievement goal theory (Elliot, 2005; Senko, 2016) and Self-Determination Theory (Ryan and Deci, 2017). More precisely, we examine whether the type of achievement goals (i.e., task or performance goals) induced via task instructions, termed the “what” of achievement goals, indeed combines with the reasons for adopting them (i.e., autonomous or controlled), termed the “why” of achievement goals, to explain students’ cheating behaviors (Vansteenkiste et al., 2014a). The study builds on goal complex literature (Senko, 2016; Sommet and Elliot, 2017), which suggests that, in order to understand fully motivational impacts on behavior, we need to consider individuals’ achievement goal pursuits and their underlying reasons for holding them. As cheating is the result, not only of immediate contextual influences, but also more long-term patterns of socialization (Pulfrey and Butera, 2013), we also examine the moderating role of individual values on cheating in these different contexts.

### Achievement Goals, “What” We Want to Achieve, and Cheating

Achievement goal theory (Urdan, 1997; Dweck, 1999; Elliot and McGregor, 2001) argues that aspirations toward competence can take different forms. People may seek to master the requirements of the task or to pursue intrapersonal improvement, what Elliot and McGregor (2001) classify as mastery-goals, or be more focused on outperforming others, achievement striving termed as performance-goals. When these achievement goals hold the promise of extending one’s competence, they have a positive valence, thereby instigating an approach orientation. However, when one is focused on avoiding incompetence, an avoidance orientation, focused on keeping failure at bay, is elicited (Elliot and McGregor, 2001).

Achievement goals have been associated with a wide range of competence-relevant outcomes, including engagement, learning strategies, and performance (see Hulleman et al., 2010; Van Yperen et al., 2015; Senko and Dawson, 2017). It is therefore not surprising that achievement goals are likely to play a key role in decisions to cheat or not (Anderman and Danner, 2008) as cheating is indeed one way to seemingly achieve socially recognized competence. In fact, both acceptance of cheating and actual cheating behavior have been positively linked with the pursuit of performance goals (Van Yperen, 2006; Van Yperen et al., 2011).

The type of achievement goal one holds is at least partially rooted in the classroom goal structures, which denotes the competence-relevant foci that are salient in the learning environment through classroom practices and communication (Murayama and Elliot, 2009). While performance goal structures emphasize the importance of demonstrating high ability through outperforming peers, mastery goal structures put the focus on developing task mastery and/or task-related self-improvement (Kaplan et al., 2002). A number of studies have found evidence for a positive association between performance classroom goal structures and positive attitudes toward cheating (Kaplan et al., 2002; Murdock et al., 2004) as well as actual cheating, albeit self-reported. To illustrate, Anderman et al. (1998) reported that, in a study carried out with middle school students, self-report cheating was positively correlated with perceptions of classroom and institutional-level performance-goal orientation. Furthermore, longitudinal results show that students transferring from less performance-oriented middle school math classes to more performance-oriented high school math classes admit to increasingly engaging in cheating (Anderman and Midgley, 2004). Murdock et al. (2007) reported a positive relation between performance-oriented classrooms and the perceived justifiability of cheating. These findings coincide with results of a meta-analysis of determinants of cheating among college students (Whitley, 1998), which found that perceived competition and greater rewards for success are robust predictors of students’ likelihood of cheating in their studies.

Conversely, research has also established a negative relation between mastery goal adoption or promotion and cheating (Jordan, 2001; Pulfrey and Butera, 2016). This relation can potentially be explained by the fact that the desire to master course material and an understanding of the importance of what is being learnt, as well as underlying values associated with mastery-goal adoption are all antithetical to cheating behavior.

While many of the studies investigating both personal and contextual achievement goals have amalgamated performance-approach (*approaching* normative competence) and performance-avoidance (*avoiding* normative incompetence) goals in analyses (e.g., Van Yperen et al., 2011), other goal research has shown that performance-*approach* goals have a particular connection with cheating (Anderman et al., 1998). Pulfrey and Butera (2013) built on this, showing that performance-approach goals mediate the relation between individual adherence to more general life values of achievement and power and the condoning of cheating. While research has also found associations between performance-avoidance goal adoption and cheating (Niiya et al., 2008, for example), in this study we focus on the role of performance- and mastery-approach goal inductions, as these are likely to be more present in task instructions in the classroom setting (Pope, 2000).

### Motivation, “Why We Want to Achieve,” and Cheating

Just as Achievement Goal Theory adopts a differentiated viewpoint toward the type of achievement goals one pursues, Self-Determination Theory argues that people can report qualitatively different types of reasons for engaging in their daily activities (Ryan and Deci, 2000). These reasons are conceptualized to fall along a continuum, stretching from a more controlled forms of regulation, in which heteronomous sources outside the self are prompting action, to more autonomous forms of regulation, in which the impulse to act is volitional and comes from within. When controlled, we act to avoid external punishment or gain externally offered rewards [external regulation (EX)] or we can act to meet internal pressures, such as feelings of shame, guilt, and blame or to bolster our ego [introjected regulation (IJ)]. When autonomously regulated, we perceive our behavior as being congruent with what we consider as personally important and valuable (identified regulation), or we derive a sense of pleasure and enjoyment from the activity [intrinsic motivation (IM)] (Ryan and Deci, 2000). These different types of regulation can be measured and an overall degree of autonomous regulation for a particular activity, the relative autonomy index (RAI), calculated using the formula: (Autonomous regulation = ((IM^∗^2)+ID)−((EX^∗^2)+IJ) (Grolnick and Ryan, 1989; Ryan and Connell, 1989).

Reasons for acting do not solely apply to our daily *behavior*, but also to the type of *achievement goals* we pursue (Vansteenkiste et al., 2014a). A goal complex involves the consideration of both the type of achievement goals people adopt (“*what*” of achievement goals) and their reasons for adopting them (“why” of achievement goals), which from a SDT-perspective can be more autonomous or more controlled. Multiple studies have shown that these underlying reasons matter above and beyond the type of achievement goals, in many cases even being a more robust predictor of outcomes as diverse as deep cognitive processing (Vansteenkiste et al., 2010b), positive affect (Gillet et al., 2012), and the use of effective learning strategies (Michou et al., 2014) and performance (Gaudreau, 2012).

The empirical work on individuals’ autonomous and controlled regulation and cheating is more limited. As for students’ motives for studying, cheating has been found to be positively associated with controlled motives, such as studying to get a well-paid job (Murdock et al., 2001; Davy et al., 2007), to gain social approval (Pulfrey and Butera, 2013), or to earn a material incentive (Anderman et al., 1998). In contrast, autonomous forms of study motivation have been shown to relate negatively to cheating (Schraw et al., 2007; Vansteenkiste et al., 2009; Orosz et al., 2013; Peled et al., 2019).

There is also some evidence that the reasons underlying achievement goals as such predict cheating. For instance, autonomous and controlled motives underlying performance-approach goals (but not the pursuit of performance-approach goal as such) were found to relate negatively and positively, respectively, to self-reported cheating in high school students (Vansteenkiste et al., 2010b). Along similar lines, in the domain of sport, adult soccer players who held more controlled reasons for pursuing performance-approach goals reported a greater tendency to depersonalize their opponents, which increased their tendency to display unfair and aggressive behavior during the game (Vansteenkiste et al., 2010a). Regarding moral behavior, Oz et al. (2016) found that cheating was also negatively related to autonomous motives underlying both performance-avoidance and approach goals.

While especially the controlled regulation of performance goals seems to increase one’s vulnerability for cheating, the combined presence of autonomous reasons underlying mastery-approach goals may restrain cheating and unethical behavior more generally. For instance, Michou et al. (2014) showed that autonomous motivation underlying mastery-approach goals was negatively related to self-reported cheating in a sample of university students. Vansteenkiste et al. (2014b) found, in a sample of youth volleyball players, that in games where the players adopted a mastery-approach goal for autonomous reasons, they reported engaging in more pro-social behavior toward teammates.

### Manipulating the “What and Why” of Goal Pursuit in Task Instructions

Much of the research into the combined approach of achievement goals and underlying motivation has been devoted to understanding why individuals cheat *from their perspective*. The question remains as to whether we can design the environment in such a way as to activate a specific achievement goal and underlying reason, with the aim of reducing cheating to a maximum. Previous experimental work in this context is relevant, which either presented a task with the aim of pursuing a task- or performance-oriented goal (e.g., Elliot and Harackiewicz, 1994; Van Yperen et al., 2015), or which made use of a more autonomy-supportive or a more controlling communication style to introduce the task (e.g., Savard et al., 2013).

Although various studies have experimentally examined both of these dimensions in isolation, only a handful of studies focused on simultaneously manipulating achievement goal contents and different communication styles when instructing participants to work on a task. Informative in this respect is work by Benita et al. (2014), who found that both achievement goals and an autonomy-supportive, relative to a controlling, style can, both independently but also in a synergistic way impact on participants’ motivation and engagement. More directly relevant to our purposes, Spray et al. (2006) found that participants in autonomy-supportive goal induction conditions persisted longer, enjoyed better, and performed higher in a task than participants in controlling goal induction conditions. Moreover, participants in a task involvement goal induction condition performed higher than those in an ego involvement goal induction condition. Sommet et al. (2019) also showed that inducing performance goals in a controlling as opposed to an autonomy-supportive way positively predicted self-reported exploitation toward information exchange and through this, negatively related to information sharing. Although these studies are informative, as they show that we can impact motivational and emotional states as well as pro-social behavior through the combination of inducing specific achievement goals in a particular way via task instructions, the question whether they would predict cheating has not been addressed, yet.

### Self-Report Cheating Versus Cheating Behavior

To date the research literature on cheating has worked extensively with self-report cheating measures (e.g., Anderman and Midgley, 2004; Vansteenkiste et al., 2009). Although some studies have shown similar patterns between self-report cheating and behavioral measures of cheating (e.g., Pulfrey and Butera, 2013), as we know from the literature on social desirability versus social utility in achievement goal adoption (see Dompnier et al., 2009), what people say may not necessarily coincide with their behavior as social desirability concerns may be operating. This problem is particularly relevant in the case of socially desirable and socially unacceptable behaviors, such as cheating. Consequently behavioral measures add value (see DeAndrea et al., 2009) as they tap into what people will actually do in the situation, bypassing potential self-censorship or image-management issues.

Hence, our aim in this research is to bring together the combined approach to motivation encapsulated in the *what* and *why* of Goal Complex Theory in an experimental approach and to test the impact of this on cheating behavior. To this end, we examine whether the way that task instructions frame the targeted achievement goal of a task (i.e., performance-approach or mastery-approach) and the way this task and achievement goal is presented (i.e., in an autonomy-supportive or controlling way) impact participants’ goals, the degree to which those goals are adopted for autonomous reasons, and the degree to which they cheat.

### The Role of Individual Values

Apart from contextual predictors of individuals’ goal adoption and underlying motives, more enduring personal characteristics, like individuals’ values, can also predict the likelihood of cheating. Values are higher-order, life goals (Schwartz, 1992, 2006) and are, to a significant extent, rooted in ongoing socialization from key social agents such as family, friends, and school or work (Kasser et al., 1995). Pulfrey and Butera (2013, 2016) and Pulfrey et al. (2018) showed that not all values are created equal when it comes to cheating. Specifically, they reported robust associations between adherence to self-enhancement values (i.e., competition, outperforming others to achieve social approval, status, power) and holding a positive attitude toward cheating as well as actual cheating behavior among university and business school students.

Interestingly, there exists evidence that individual values also interact with more immediate contextual influences in the determination of both attitudes toward cheating and cheating behavior. For instance, adherence to self-enhancement values has been shown to interact with competitive versus cooperative contextual primes, with those higher in self-enhancement value adherence more likely to openly condone cheating in a competitive environment, exemplified by the description of society as a competitive, free market (Pulfrey and Butera, 2013), than in a cooperative one. Pulfrey et al. (2018) also showed that those who were willing to engage in cheating in the company of a relative stranger, a risky enterprise as it is harder to predict the reaction of the other, were also higher on self-enhancement value adherence. These results imply that individual values, the result of long-term socialization, are likely to have an impact on cheating behavior and also to act in interaction with contextual primes that emphasize performance versus mastery goals.

### Present Study

In the present study, we extend the available work at the intersection of Achievement Goal Theory and SDT (Vansteenkiste et al., 2014a) by investigating how the combination of an experimentally induced achievement goal and a specific style of introducing the achievement goal influence objectively measured cheating behavior. Specifically, we investigate whether tasks presented as serving the pursuit of either performance-approach or mastery-approach goals and an autonomy-supportive versus controlling style of presenting the task and achievement goal influence participants’ cheating behavior. We hypothesize that the combination of promoting mastery-approach goals in an autonomy-supportive way may offset the inclination to cheat (Hypothesis 1).

Apart from these contextual predictors, we also considered the role of self-enhancement values (achievement, power dominance and resources, and face saving/image), both in isolation as well as in combination with the experimental manipulations. Specifically, according to previous findings reported above, we hypothesize that self-enhancement values will predict cheating behavior (Hypothesis 2a; main effect) and may interact with the experimental manipulations in the prediction of cheating (Hypothesis 2b; moderation effect) behavior. Specifically, the presumed cheating-inhibitory benefits of specific goal complexes (i.e., task-approach/autonomy-support) may be attenuated among those endorsing self-enhancement values, while the elevated vulnerability for cheating in other goal complexes (i.e., performance-approach/control) would be more strongly activated among those holding self-enhancement values.

We tested these hypotheses in two experimental studies using the same task instructions and experimental conditions in two different contexts. In Study 1, the experiment took place in classroom environment. In Study 2, the experiment took place in a laboratory.

## Study 1

The aim of Study 1 was to test these hypotheses in an experiment that was carried out in the ecologically rich environment of a real classroom setting.

### Materials and Methods

#### Participants

One hundred and sixty-four second-year students attending an international management school based in Switzerland, with a mean age of 20.43 (*SD* = 1.50) years, participated in this study. The sample consisted of 87 male and 73 female students.

#### Procedure

A visiting researcher carried out the study in the students’ Organizational Behavior class (a 90′ weekly class) and its two phases were presented as two separate studies, the first as an investigation into what students think about life and work and the second as part of a research program on spatial exercises. Participation was entirely voluntary and any student who did not wish to participate was given the opportunity to get on with course work. All students present in all the classes verbally expressed their desire to participate. One questionnaire was returned not having been filled in. Prior to commencing the researcher introduced the phases and, using a short power-point presentation, explained the full procedure of both, including the fact that, in the spatial exercise testing, there would be two sets of timed puzzles to do (see Pulfrey and Butera, 2013) and instructions on how to do the puzzles. We provided two sets of exercises as mastery-approach intrapersonal improvement goals focus on improving personal performance, so this provided participants the chance to have a go in the first set and then try to do better in the second. In the first phase prior to the spatial exercise, there was the values questionnaire. This part was anonymous. Then, when everyone had finished, the second phase (i.e., the experimental manipulation), which ostensibly came from a different research department, was launched.

#### Experimental Induction

To test our key hypotheses, four experimental conditions were operationalized by means of introductory texts written on the first page of the second phase. These texts (see the Appendix for full texts) prompted the adoption of either a mastery-approach goal focused on intrapersonal improvement (“*… achievement is all about personal improvement … work individually trying to improve your personal performance*”) or performance-approach goals (“*… achievement is all about who does best … work individually … trying to perform better than the other students.*) (Murayama and Elliot, 2009), in combination with either a controlling (“*… test, which evaluates your capacity*,” “*look upon this task as a way of impressing others*,” “you must”), or autonomy-supportive (“*… exercises, which most students find an interesting challenge*,” “*look upon this task as a personal challenge*,” “you can”) style of introducing the task and achievement goal. The students were distributed in six sections for their Organizational Behavior class and so questionnaires were randomly distributed to ensure an even spread of the conditions in each class: controlling/performance (CP) condition (*n* = 40), controlling/mastery (CM) condition (*n* = 42), autonomy-support/performance (AP) condition (*n* = 42), and autonomy-support/mastery condition (AM) (*n* = 40) [χ^2^(1, *N* = 164) = 8.71, *n.s*.]. In order to increase classroom validity and motivation to take the activity seriously, we informed participants that they would receive feedback on their performance once the study was completed and so they were required to put their names on this test. As the first values survey was supposed to be anonymous, 14 digit “patent numbers” in gray print, positioned very discretely at the bottom of the page of both the values survey and the diagnostic test were used to couple each student’s two documents if necessary. On the first page, there was also a space to fill in the participant’s name and year of study and on the second page, a sample special exercise presented. Participants were informed orally and in writing that they had 8 min to solve a first set of special exercises presented from the third page on.

As in Lobel and Levanon (1988) and Pulfrey and Butera’s (2013) research paradigm, in the special exercises, participants were asked to draw figures without lifting their pencil off the paper and without retracing any line. For three of the problems in each set of exercises, this was possible but for the other three this was impossible, even though the figures did not ostensibly look more complicated than the solvable ones. Participants were given a space to practice their drawings in and a box below this in which they were instructed to draw their solution *only if* they had succeeded in solving the problem.

After the 8 min had expired, all participants had to stop and on a separate page they were asked the question: “Which exercises did you succeed in completing?” Following this were six affirmations: “I was able to do exercise 1, 2, 3” etcetera and for each affirmation participants ticked a box marked yes or no. They then read a short text reminding them of their goal in either a controlling or autonomy-supportive manner, according to the condition they were in and then completed the second set of exercises, which was the same as set one but with different figures. Finally, they filled in a series of motivation and goal-related questions, including a manipulation check which asked them to state the goal that they had been pursuing and the reason for the goal. Students were debriefed once all the data had been collected. No indications of suspicion about the cover story or about the impossible nature of some of the figures were observed.

#### Measures

##### Schwartz value survey

The students’ individual values were measured using the refined Schwartz values questionnaire (Schwartz et al., 2012). Scale reliabilities for the four value types were satisfactory: self-enhancement (achievement, power dominance and resources, and face saving/image) (α = 0.82); self-transcendence (α = 0.76); open to change (α = 0.81); conservation (α = 0.83). The self-enhancement score was centered around the mean of all values as suggested by Schwartz et al. (2012), and ranged from −1.98 to 1.23 (*M* = −0.17, *SD* = 0.59).

##### Cheating

Cheating was calculated on a count basis (0–3 for each set of exercises). We considered that participants cheated when they “solved” at least one of the impossible problems *and* ticked the relevant “Yes, I have solved the problem” box on the final page of set of each exercises. For the sample as a whole, 51 participants (32.48%) cheated (16 in the CP condition, 13 in the CM condition, 14 in the AP condition, and 8 in the AM condition).

##### Manipulation check

The literature on goal manipulation has often included manipulation checks in order to assess whether participants had actually understood and taken in the goal-related instructions as well as to be able to affirm that they were acting under the conscious influence of the goals they had been assigned (e.g., Spray et al., 2006; Van Yperen et al., 2009). After finishing the exercises and reporting their results, participants filled in goal check questions (Elliot et al., 2011): In these exercises my goal was to: “*Do better than other students on these exercises*” (performance-approach), “*Do better as I go through them*” (mastery-approach), They responded on a seven-point scale going from 1 (totally disagree) to 7 (totally agree). We then repeated the two options and asked participants to indicate the goal that was most important to them. After this we instructed participants to think about why they wanted to achieve the goal they had chosen. Working out from the original Ryan and Connell (1989) Self-Regulation Questionnaire, we then asked participants to rate their degree of agreement with the following four statements tapping into participants’ reasons for pursuing an achievement goal: I have to comply with the demands of others (extrinsic motivation with EX), I would feel bad, guilty, or anxious if I didn’t (extrinsic motivation with IJ), I find this a personally valuable goal [extrinsic motivation with identified regulation (ID)], I find this a highly stimulating and challenging goal (IM). The four goal motivation statements were used to create a single autonomy index, using the formula: (Autonomous motivation for goal adoption = ((IM ^∗^ 2) + ID) − ((EX ^∗^ 2) + IJ) (Ryan and Connell, 1989). We expected participants in the two mastery conditions to endorse the mastery goals and those in the performance conditions to endorse the performance goals. In like vein, we expected participants in the controlling conditions to express a more controlled motivation underlying the endorsed goal and those in the two autonomy-supportive conditions to express a more autonomous motivation underlying the endorsed goal. See Table 1 for descriptive statistics.

**TABLE 1:** Study 1 (*N* = 164): descriptive statistics.

	***M***	***SD***	**Range**	**(1)**	**(2)**	**(3)**	**(4)**
Relative adherence to self-enhancement values (1)	−0.18	0.59	−1.97–1.23	1.00			
Post-task mastery-approach goals (2)	4.84	1.69	1–7	0.13	1.00		
Post-task performance-approach goals (3)	3.79	1.83	1–7	0.30^∗∗∗^	0.42^∗∗∗^	1.00	
Pre-task autonomous reason for adopting goal (4)	5.14	1.42	1–7	−0.14	0.13	−0.14	1.00

*^∗∗∗^ denotes the significance of the *p-*values.*

#### Data Analysis

As in previous studies on cheating (Pulfrey and Butera, 2013), we used Poisson regression featuring robust standard errors to control for violation of the assumption that dependent variable variance equals its mean (Cameron and Trivedi, 2009) to test our hypothesis that promoting mastery-approach in an autonomy-supportive way would engender less cheating than promoting the same goals in a controlling way or promoting performance-approach goals in either way. The dependent variable was a count measure of acts of cheating. Our regression model included three orthogonal contrasts to enable us to compare the AM condition to the other three. The first contrast, the AM *contrast*, had the AM coded 3 and the other three conditions (CM, AP, and CP) coded −1. In the second contrast, the *AP* contrast, the AM condition was coded 0, the AP condition was coded 2, and the two controlling conditions coded −1. In the third contrast, the CM contrast, the AM and AP conditions were coded 0, the CP condition was coded −1, and the CM condition was coded 1. Self-enhancement relative individual value adherence was included in the model as previous research (Pulfrey and Butera, 2013) has shown it to be a significant predictor of cheating behavior and interactions between self-enhancement value-adherence and the three experimental condition contrasts were included. Gender was included as a control variable as previous research consistently shows gender effects with girls cheating less than boys (Whitley et al., 1999). Finally, the students’ class was reference coded with class 1 as the reference class and entered as a statistical control. This was because class 1 differed from the other classes in terms of procedure in that, in order to fit in with the teacher’s lesson plan, the researcher carried out the experimental exercise in the second part of the two-period class, whereas in the other five classes it was carried out in the first part of the double-period class. Initial analyses using a dichotomous (yes, no) measure of cheating did not indicate significant differences in levels of cheating among classes [χ^2^(1, *N* = 164) = 9.06, *n.s*.] but analyses using a count measure of cheating indicated a marginal difference [χ^2^(1, *N* = 164) = 50.12, *p* < 0.06]. Multi-level modeling was not, however, indicated in this case for three reasons. First because there were only six classes, secondly, as questionnaires were distributed randomly within classes [χ^2^(1, *N* = 164) = 8.71, *n.s*.], and thirdly, as the class-related design effect sizes for cheating in set 1 (*DEFF* = 1.13) and total cheating (in set 1 and set 2 of the exercises) (*DEFF* = 1.86) were below the cut-off point of 2 which indicates significant random contribution of higher level variables.^1^ Hence, the model contained a total of 12 predictor terms, the three contrasts, self-enhancement value adherence, interactions between the three contrasts, and values, gender, and the dummy variables for class. We carried out analyses using STATA 14.2.

### Results

#### Manipulation Checks

Testing the regression model on the intended manipulation checks, which were positioned at the end of the whole task, produced no significant results of condition. However, self-enhancement value adherence was positively related to the check question: In these exercises my goal was to … *Do better than other students on these exercises* (performance-approach), *B* = 1.23, *SE* = 0.55, *t* = −2.26, *p* = 0.03. The more participants adhered to self-enhancement values, the more they affirmed having adopted a competitive goal.

#### Hypothesis 1 *–* Effect of Experimental Manipulations

**FIGURE 1:**
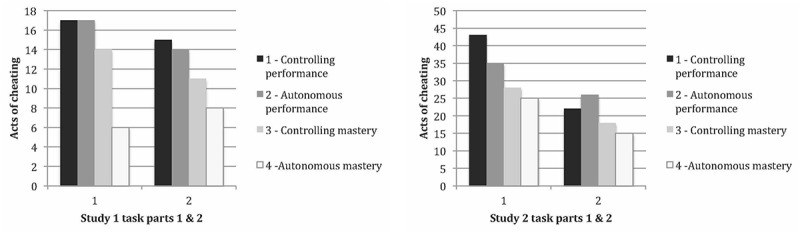
Studies 1 and 2: number of acts of cheating per condition.

Results revealed a main effect of contrast one, the AM contrast on cheating in the first set of exercises, *B* = −0.35, *SE* = 0.14, *z* = −2.54, *p* = 0.01. Testing relations between individual conditions, we found that the AM produced significantly less cheating than all three other conditions; the CM condition, *B* = −1.45, *SE* = 0.61, *z* = −2.37, *p* = 0.02; the AP condition, *B* = −1.28, *SE* = 0.64, *z* = −2.02, *p* = 0.04; the CP condition, *B* = −1.41, *SE* = 0.58, *z* = −2.41, *p* = 0.02. When it came to cheating in the second set of exercises, there were no main effects. Aggregating cheating counts from set 1 and set 2 of exercises allowed us to test the model with the total amount of cheating from sets 1 and 2 combined. Results revealed a significant effect of contrast one, the AM contrast on overall rates of cheating (exercise one and two combined), *B* = −0.20, *SE* = 0.09, *z* = −2.12, *p* = 0.03. There were no other significant effects. See Figure 1 for a comparative view of cheating in the four conditions.

#### Hypothesis 2 – The Role of Self-Enhancement Values

**FIGURE 2:**
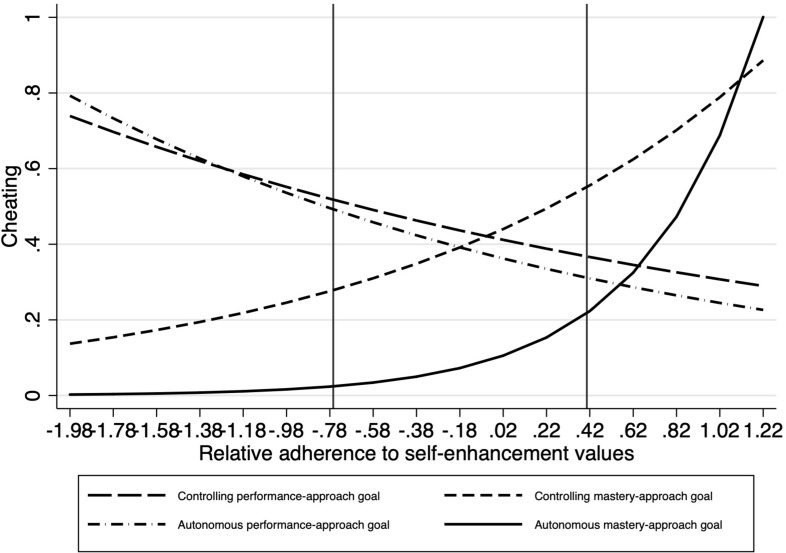
Study 1: impact of interaction between relative adherence to self-enhancement values and experimental condition on cheating in part 1 of the exercise [cheating increases with relative adherence to self-enhancement values in the autonomous-mastery condition and there is less cheating in the autonomous mastery condition compared with the controlling performance-approach condition at low levels (–1 SD) of self-enhancement].

In set 1 of the exercises, we found an interaction effect between the AM contrast and relative self-enhancement value adherence, *B* = 0.48, *SE* = 0.22, *z* = 2.14, *p* = 0.03. In terms of simple effects, cheating increased with relative adherence to self-enhancement values in the AM condition, *B* = 1.82, *SE* = 0.81, *z* = 2.26, *p* = 0.02. There was also a significant difference between amounts of cheating in this condition compared with the CP condition at *low* levels of self-enhancement, −1 *SD*, *F*(1,159) = 6.32, *p* = 0.01, with less cheating in the AM (Figure 2).

**FIGURE 3:**
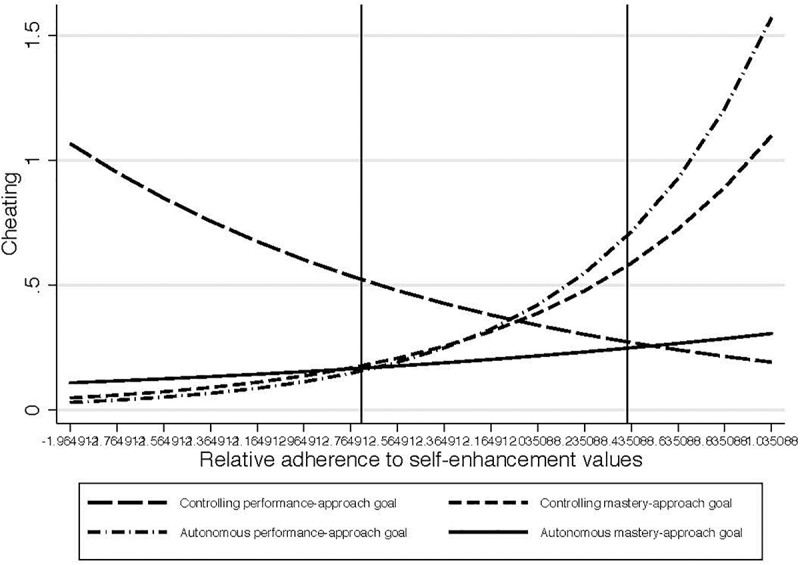
Study 1: impact of interaction between relative adherence to self-enhancement values and experimental condition on cheating in set 2 of the exercise (cheating increases with self-enhancement value adherence in the *autonomous-performance* condition).

In set 2, there was an interaction effect between the self-enhancement value adherence and the CM contrast, *B* = 0.95, *SE* = 0.38, *z* = 2.57, *p* = 0.01. Here cheating increased with rising relative adherence to self-enhancement values of power, achievement, and face in the *AP condition*, *B* = 1.19, *SE* = 0.57, *z* = 2.11, *p* = 0.04 (Figure 3).

#### Supplementary Analyses

In order to see if the result of less cheating in the AM condition compared with the others could be due to strategic cheating, that it to say not cheating in set 1, then cheating in set 2 to ensure a better result as instructed in the mastery conditions, we tested the model on cheating in set 2, for the 122 participants who had not cheated in set 1 (CP condition, *N* = 29; CM condition, *N* = 30; AP condition, *N* = 31; AM condition, *N* = 33). Results revealed no main effects differences between conditions.

### Brief Discussion

Study 1 tested the impact of task instructions, emphasizing either the pursuit of performance or mastery-approach goals introduced in either a controlling or autonomy-supportive way, on student cheating in an experiment carried out collectively in a classroom context. Results revealed lower levels of cheating in the first set of the exercise in the AM condition compared with all the other three conditions. There were also lower levels of cheating in this condition compared with the other three conditions combined in the task as a whole, although not all the simple effect comparisons were significant. This indicates that, as predicted, the mere fact of focusing in the written task introduction on mastery-approach goals of interpersonal improvement in an autonomy-supportive way was sufficient to provoke less unethical behavior in the task compared with the other conditions. On the level of goal complex theorizing, this result adds experimental, behavioral support to the numerous studies that indicate that both goal content and the reason behind the goal adoption seem to be important in determining behavior (e.g., Vansteenkiste et al., 2010a). Here we see that promoting mastery-goals is beneficial in encouraging honest behavior, but above all when they are promoted in an autonomy-supportive way. On the more practical level, considering the potentially low level of attention students in a classroom context might pay to introductory blurb to an exercise, this result points to the potential importance and efficacy of this sort of task-introduction, if we wish to work actively against cheating. However, the fact that this effect was only significant in the first set of exercises alerts us to the fact that it is comparable to a priming effect, which notably has a short duration of efficacy.

This conclusion is supported by the lack of impact of the experimental manipulations on the supposed manipulation check (see note 3). This lack of any significant effects on self-report goals and reasons for adopting these goals can be explained by two potential reasons. Firstly, as we put the measures at the end of the task it is possible that the priming effect of the condition instructions had worn off by the end of the task, particularly as participants were very engaged in the exercises. Secondly, we asked participants to report their own goals, as opposed to the goals that they had been set and prior research has clearly indicated that a strong social desirability bias can prevent the majority of students from owning up to a competitive goal, as learning goals are deemed more socially acceptable (Darnon et al., 2009). The fact that self-enhancement value adherence predicted performance-approach goal adherence (Likert scale question), but did not prioritize it over mastery-approach goal adherence (forced choice question), supports these explanations.

The interaction effects with relative adherence to self-enhancement values intimate that self-enhancing individuals may be less susceptible to autonomy-supportive contextual cues. The autonomy-supportive/mastery goal instructions are particularly efficient for those low in self-enhancement (as shown by there being less cheating in the AM than in the CP conditions) and adherence to self-enhancement values relative to other values predicts cheating in the autonomy-supportive/mastery condition. The graphing of this effect indicates that it is indeed the “hard-core” believers in power, face, and publically acclaimed achievement that still cheat in this condition.

## Study 2

In spite of the promising results obtained in Study 1, we decided to try to replicate the findings in a second study. In doing so, we made three significant adaptations to the first study. First, to see if the result obtained in Study 1 might be reproduced in a laboratory context where participants might be less distracted by external factors (e.g., their classmates, social pressure) implicit environmental primes relating to the class, the classroom, and/or classmates as well as potential autobiographical, classroom priming effects. Second, in order to add further to the generalizability of our findings, we drew our sample from a mixed faculty population, as business school students have been found to have more lax attitudes to cheating than students from other faculties (Klein et al., 2007). Third, given the lack of clear effects on our manipulation check procedures in Study 1, we refined our manipulation check procedure to be sure that the effects found in Study 1 were indeed condition-related. To sum up, the aim of Study 2 was to re-test our hypotheses in order to see if the pattern of cheating would replicate in a different task context.

### Materials and Methods

#### Participants

One hundred and sixty students attending a Swiss public university, with a mean age of 22.32 (*SD* = 2.97) years, participated in this study. The sample consisted of 80 male and 80 female students. The participants were recruited by the Faculty of Business and Economics experiment recruitment scheme and were paid 10 Swiss francs for participating in the study. Participants were recruited who spoke and understood English and had confirmed that they had a good level of understanding of spoken English prior to attending the experiment. Thirty-five percent of the sample came from the Faculty of Business and Economics and the rest of the sample was a spread from other faculties, including social sciences, humanities, engineering, and pure science. All participants signed a consent form prior to starting the experiment.

#### Procedure

The study was carried out in the Faculty of Business and Economics experimental laboratories, in a room containing a minimum of 10 individual cubicles each with a computer in it. A trained research assistant welcomed groups of approximately 10 participants at a time, explained the protocol, and assigned the students each to an individual cubicle. Participants signed a consent form and then everything else happened online in the context of an online questionnaire. Participants first of all filled in the preliminary pre-task questionnaire (individual values; Schwartz et al., 2012). Following this, they read the instructions to the task, then the introductory text, which was in fact the experimental induction. After this they responded to the first experimental manipulation check, a *pre-task* manipulation check. Participants responded to the first set of task questions. Then in-between the first and second set of task questions, they filled in a second manipulation check, the *mid-task* check. They then did the second part of the task. Once they had completed the task, they filled in the third manipulation check, the *post-task* manipulation check. When they had finished the research assistant thanked and paid them 10 Swiss francs. The experiment lasted in total about 20 min and was completely anonymous. No indications of suspicion about the impossible nature of some of the puzzles were observed.

#### Experimental Induction

As in Experiment 1, to test our hypotheses, four experimental conditions were operationalized by means of same introductory texts as in Study 1 (see the Appendix). Participants were randomly assigned to one of the four conditions with 40 participants in each condition. Once they had filled in the preliminary questionnaire (individual values; Schwartz et al., 2012), they read the instructions to the task, followed by the introductory text.

Following this, they responded to the first experimental manipulation check, a pre-task check. This consisted of two questions asking them what goal they had been instructed to follow and then four questions with different reasons for pursuing this goal. Then they started the task, which was presented as an evaluation of working memory capacity. The task involved listening to 24 working memory questions taken from the WJ_IV COG Test 3 – verbal attention test (Schrank and Wendling, 2018) divided into two sets. In each question participants firstly heard a person saying names of animals and numbers, for example: “9 rabbit 5 4 goose”. Following each question they heard the speaker asking them to write down some of the names and numbers mentioned in the question in a space provided, for example: “Which was the number before rabbit and the last animal mentioned?” Participants were clearly informed that they should listen to the list, listen to the question, and then write down their answer. They had a time limit for writing down each answer and were informed that they would only have time to listen to the question once and write down the answer before the test progressed automatically to the next question on a new page.

The original test, which progresses from easy to difficult, was adapted in two ways. Firstly, to test cheating, we created four combined questions, that generated a load that was too great for the working memory to handle. For example, participants heard, “9 rabbit 5 4 goose chicken 3 horse 7 kitten … beep … . Tell me the first and the last animal.” As working memory capacity is restricted to three to five chunks (Cowan, 2010), the only way to succeed in these questions was by cheating, in other words writing down the words and numbers as they were spoken and then erasing those that were not required in the answer. As the test was presented as a test of working memory, to allay potential suspicions that there were impossible questions, participants were told in the instructions: “You might find that some questions seem quite difficult. The test is meant to be challenging.” The double questions were spaced out in the test, Q. 6, 9, 15, and 23. As the early questions are very simple in the original test, we placed one normal but longer question as Q. 3 to alert participants to the fact that they would not find all the questions easy and thus to encourage those who might be willing to cheat to develop a strategy.

#### Measures

##### Schwartz value survey

As in Study 1 the same Schwartz Values Questionnaire (Schwartz et al., 2012) was used to assess participants’ values. Scale reliabilities for the four value types were once again satisfactory: self-enhancement (α = 0.84); self-transcendence (α = 0.83); open to change (α = 0.83); conservation (α = 0.84). The range of the self-enhancement score centered round the mean of all values was from −1.96 to 1.14 (*M* = −0.51, *SD* = 0.57).

##### Cheating

Cheating was again calculated on a count basis. We considered that participants cheated when they “solved” at least one of the impossible questions. For the sample as a whole, 92 (63.12%) cheated (31 in the CP condition, 24 in the CM condition, 23 in the AP condition, and 23 in the AM condition).

##### Manipulation check

In order to check if participants had read and understood the instructions, the first check was positioned immediately after the experimental induction in the introduction to the task and consisted of two goal check questions (Elliot et al., 2011): “In this task, the main goal I have been set is *… to do better than other students on the exercises* (performance-other-approach (PA) and … *to do better in the second set of exercises than in the first* (mastery-self-approach (MA).” These were followed by four goal reason questions, which again tested the four different types of motivational regulation: (1) Extrinsic motivation with EX: “Why will you pursue this goal*? … It’s part of what I have to do for the experiment*, (2) Extrinsic motivation with IJ: *I want people to have a good opinion of me*, (3) Extrinsic motivation with identified regulation (ID): *I think it’s useful*, (4) IM: *It’ll be interesting*. Participants responded on a scale of 1–7, ranging from “not at all” to “yes, absolutely.” As in Study 1 and similar to prior studies (e.g., Vansteenkiste et al., 2005), we created a RAI score by weighting the four goal reasons congruent with their positioning on the self-determination theory continuum [(IM ^∗^ 2) + (ID) − (IJ) + (EX ^∗^ 2)] and used this as the manipulation check.

There was a second check positioned between part 1 and part 2 of the exercises. This was a multiple-choice question with two options: “What is the goal you were asked to achieve as you do the exercises? … *Outperform other students and be the best*” (PA) or “*Do better in the second set of exercises than the first*” (MA). Again this was followed by four goal reason questions: “Why are you pursuing this goal?” with the same reasons as above. The third manipulation check positioned at the end of the test. This was consisted of the two 3-item scales of mastery-approach, intrapersonal improvement (α = 0.97) and performance-approach (α = 0.97) goals developed by Elliot et al. (2011), introduced by the phrase: “In this task, the main goal I had was …”. This was followed by the same goal reason questions as above. Participants responded on a scale of 1 (not at all) to 7 (yes, absolutely).

#### Data Analysis

As in Study 1, we used Poisson regression, with the robust standard error option to test our hypothesis that promoting mastery-approach interpersonal improvement goals in an autonomy-supportive way would engender less cheating that promoting the same goals in a controlling way or promoting performance-approach goals. Our regression model was the same as in Study 1 and included the same orthogonal contrasts,^2^ the AM contrast (AM = 3, the other conditions = −1), the *AP* contrast (AP = 2, AM = 0, the other conditions = −1), and the CM contrast (CP = −1, CM = 1, the other conditions = 0). Self-enhancement relative individual value adherence was again included in the model and interactions between self-enhancement value-adherence and the three experimental condition contrasts were included. Gender was again included as a control variable and also faculty as previous research indicates that business school students have more lax attitudes toward cheating (Klein et al., 2007). Hence, the model contained a total of 12 predictor terms.

### Results

Descriptive analyses and correlations of the variables are presented in Tables 2, 3.

**TABLE 2:** Study 2 (*N* = 160): descriptive statistics – means.

	***M***	***SD***	**Min.**	**Max.**
Relative adherence to self-enhancement values	–0.51	0.57	–1.96	1.14
Pre-task mastery-approach goals	4.61	2.11	1.00	7.00
Pre-task performance-approach goals	4.37	2.12	1.00	7.00
Pre-task autonomous reasons	24.47	3.95	12.00	33.00
Mid-task autonomous reasons	6.83	5.59	–10.00	21.00
Post-task autonomous reasons	7.29	4.80	–10.00	20.00
Post-task mastery-approach goals	4.94	1.72	1.00	7.00
Post-task performance-approach goals	4.03	1.97	1.00	7.00

**TABLE 3:** Study 2 (*N* = 160): descriptive statistics – correlations.

	**(1)**	**(2)**	**(3)**	**(4)**	**(5)**	**(6)**	**(7)**	**(8)**
Relative adherence to self-enhancement values (1)	1.00							
Pre-task mastery-approach goals (2)	0.04	1.00						
Pre-task performance-approach goals (3)	0.05	–0.41^∗∗∗^	1.00					
Pre-task autonomous reasons (4)	–0.05	0.16^∗^	–0.06	1.00				
Mid-task autonomous reasons (5)	0.06	0.13	–0.07	0.16^∗^	1.00			
Post-task autonomous reasons (6)	0.05	0.08	0.04	0.25^∗∗^	0.76^∗∗∗^	1.00		
Post-task mastery-approach goals (7)	0.08	0.62^∗∗∗^	–0.45^∗∗∗^	0.17^∗^	0.18^∗^	0.14	1.00	
Post-task performance-approach goals (8)	0.16^∗^	–0.51^∗∗∗^	0.73^∗∗∗^	–0.12	0.03	0.11	–0.42^∗∗∗^	1.00

*^∗^, ^∗∗^, and ^∗∗∗^ denotes the significance of the *p-*values.*

#### The Manipulation Checks

Results of the first pre-task manipulation check, run with a linear regression model comparing the two mastery-approach conditions (controlling and autonomy-supportive) with the two performance-approach conditions, revealed that participants in the two mastery conditions indeed indicated that they had been set a mastery-approach goal, *B* = 2.48, *F*(1,158), *t* = −84.02, *p* = 0.000. Participants in the two performance conditions also indicated that they had been set a performance-approach goal, *B* = 2.73, *F*(1,158), *t* = 113.21, *p* = 0.000. In the second check we ran a regression to compare the goal reason in the two autonomy-supportive conditions with the two controlling conditions. To do this we created a RAI score from the four goal rationale questions [(IM^∗^2)+(ID)−(IJ)+(EX^∗^2)] (Grolnick and Ryan, 1989) and used this as the dependent variable. Participants in the two autonomy-supportive conditions expressed significantly more adherence to autonomous reasons for adopting their set goals than participants in the two controlling conditions, *B* = 1.97, *F*(1,158), *t* = 10.63, *p* = 0.001.

Results of the second manipulation check held after the first set of the task and done with a logistic regression as the dependent variable was binary, indicated once again that participants in the mastery conditions affirmed that their set goal was a mastery goal to a much greater extent than those in the performance conditions, *B* = 3.66, *SE* = 0.48, *z* = 7.60, *p* = 0.000. Using the RAI index did not show a significant difference between the controlling versus autonomy-supportive conditions, but a breakdown of the RAI into the four types of motivation explained why. Namely, participants in the two autonomy-supportive conditions compared with those in the controlling conditions, expressed significantly more intrinsically motivated reasons for adopting their set goals [IM, *B* = 0.51, *F*(1,158), *t* = 4.11, *p* = 0.04, marginally more identified regulation, ID, *B* = 0.44, *F*(1,158), *t* = 3.06, *p* = 0.08] and significantly less EX [EXT, *B* = −2.26, *F*(1,158), *t* = 56.40, *p* = 0.000]. However, there were no significant differences between the autonomy-supportive and controlled groups in terms of IJ.

Results of the third manipulation check held at the end of the task and done with a linear regression as we used the mastery-self-approach and performance-other-approach scales from the Elliot et al. (2011) Achievement Goal Questionnaire revealed the same pattern. Once again, participants in the mastery conditions affirmed that their set goal was a mastery goal to a much greater extent than those in the performance conditions, *B* = 1.71, *F*(1,158), *t* = 52.71, *p* = 0.00, and participants in the performance conditions affirmed that their set goal was a performance goal, *B* = 2.27, *F*(1,158), *t* = 79.57, *p* = 0.00. There were, however, no significant differences between the autonomy-supportive and controlling conditions in terms of autonomous motivation for the task.

#### Results – Hypothesis 1 – Effect of Experimental Manipulations

Results revealed a main effect of contrast one, the AM contrast on cheating in the first set of exercises, *B* = −0.11, *SE* = 0.06, *z* = −1.99, *p* = 0.047. No other significant effects emerged. When it came to cheating in the second exercise and total cheating, there were no significant effects of condition (see Figure 1 for a comparative view of cheating in the four conditions).

#### Results – Hypothesis 2 – The Role of Self-Enhancement Values

There were no effects of self-enhancement value adoption on cheating in part 1 of the exercises, but a main effect of self-enhancement values in part 2 of the exercises, *B* = 0.10, *SE* = 0.04, *z* = 2.38, *p* = 0.02. When we tested the aggregate total score of cheating in parts 1 and 2 combined, this main effect of self-enhancement value adherence, *B* = 0.07, *SE* = 0.03, *z* = 2.03, *p* = 0.04 also emerged. Testing cheating in part 2 for those who had not cheated in part 1 also revealed a main effect of self-enhancement values, *B* = 0.10, *SE* = 0.30, *z* = 3.21, *p* = 0.00.

#### Supplementary Analysis

Once again, we tested the model on cheating in part 2, for the 68 participants who had not cheated in part 1 (condition 1, *N* = 12; condition 2, *N* = 18; condition 3, *N* = 19; condition 4, *N* = 19). Results revealed a main effect of self-enhancement value adherence on cheating, *B* = 0.21, *SE* = 0.88, *z* = 2.39, *p* = 0.02, and one main effect of condition, with more cheating in the CP condition compared with the AP condition, *B* = 1.64, *SE* = 0.83, *z* = −1.98, *p* = 0.048.

In part 2, we found a main effect of gender, *B* = −0.13, *SE* = 0.05, *z* = −2.78, *p* = 0.01, with women cheating less than men and we saw the same pattern in the total cheating score, *B* = −0.13, *SE* = 0.05, *z* = −2.78, *p* = 0.01.

**FIGURE 4:**
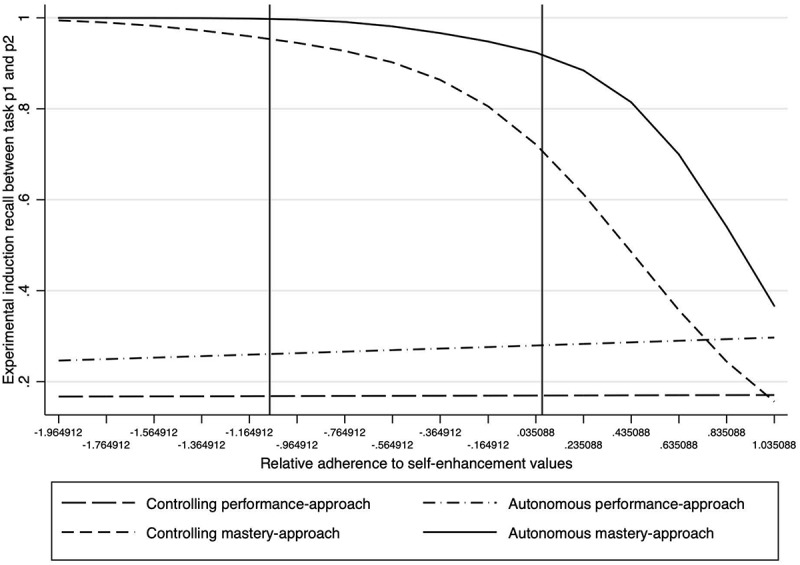
Study 2: impact of self-enhancement value adherence on participant recall of the experimental condition goal-orientation they had been set at the beginning of the task (0 = recalling the task instructions as performance-approach oriented; 1 = recalling the task instructions as mastery-approach oriented).

To understand more about why we found a main effect of self-enhancement value adherence on cheating in part 2 of the exercises and no interaction effects, we ran a supplementary analysis, testing the model on participant recall of which condition they were in mid-way through the task. To do this we regressed participant responses to the goal manipulation check question placed after the first part of the exercises (‘What is the goal you were asked to achieve as you do the exercises? … *Outperform other students and be the best*’ (PA) or ‘*Do better in the second set of exercises than the first*’ (MA).” Results of a logistic regression revealed a main effect of self-enhancement value adherence on the “recall” of having been set mastery- versus performance-approach goal in the task, *B* = 0.26, *SE* = 0.77, *z* = −3.40, *p* = 0.001, indicating that the more participants adhered to these values the more they “remembered” being instructed to outperform others generally. This supposition was supported by an interaction effect between self-enhancement value adherence and the two mastery conditions, *B* = −0.1.5, *SE* = 0.60, *z* = −2.44, *p* = 0.02, which indicated that in these two conditions, the more participants’ self-enhancement value adherence increased, the more they were likely to mis-recall the experimental instructions, thinking that they had been instructed to do better than the other participants (Figure 4).

### Brief Discussion

Using a more heterogeneous group of participants, a laboratory instead of a real-life setting, and a different task, Study 2 largely replicated the core findings of Study 1. That is, the same effect of the AM condition, involving a combination of inducing mastery-approach goals in an autonomy-supportive way, reduced cheating in the first part of the task. As in Study 1, we also see that this effect only held for the first part of the task, although, unlike Study 1, we did see a main effect for all participants, including those high on self-enhancement. However, in part 2 of the task and in the task as a whole, more deep-rooted demographic variables related to cheating, notably self-enhancement value adherence and gender took over to predict cheating with main effects and no interactions. This reinforces the argument that goal and autonomy-support versus controlling task instructions may have a priming type impact influencing behavior in the time-span immediately following their delivery, but that, like implicit priming (Shanks et al., 2013) the impact is transitory and deeper-rooted motivational influences can easily take the upper hand, once the individual is immersed in the task. The fact that self-enhancers in the mastery conditions were more likely to falsely remember the experimental induction as one setting competitive goals indicates the power of these deeper-rooted motivational influences, that can even go so far as influencing memory. We see this power also with those who had resisted the temptation to cheat in part 1 of the task as, again, it is self-enhancement value adherence that predicted giving in to the temptation to cheat, when faced with difficulties and potential threats to self-competence-related esteem in the first part of the task. The fact that those in the CP condition also gave in more than those in the AP condition implies that pressure to shine from within or without can make us more vulnerable to unethical behavior, when faced with tasks that we cannot always handle.

## General Discussion

Drawing upon two dominant motivational frameworks in the contemporary motivational landscape, that is, achievement goal theory and self-determination theory, the aim of this research was to investigate how the presentation of tasks with different goals (performance versus mastery) and different styles (autonomy-supportive versus controlling), influences behavioral cheating. More specifically, we asked the question: do the goal content and goal style when experimentally varied in task instructions both contribute to predict greater or lesser amounts of task cheating? Our core hypothesis was that task instructions that promote mastery-approach goals in an autonomy-supportive way will provoke less cheating than task instructions that promote performance-approach goals in the same way or mastery- and performance-approach goals in a controlling way. We also hypothesized that self-enhancement values will be positively related to cheating behavior and may interact with the goal induction and style of induction in the prediction of cheating. Two experimental studies in different contexts with students in higher education from different faculties provided behavioral evidence that something as minimal as the way a task is presented can indeed have an immediate significant impact on the actual amount of cheating students engage in while more stable personal attributes such as self-enhancement value adherence and gender play also an important role in cheating behavior.

### Goal Complexes Matter

Results of Study 1, which was set in a classroom context in a higher education management school, revealed firstly that participants who read instructions to a spatial exercise task that were mastery-approach focused with an autonomy-supportive style cheated less in the first part of the task than participants in conditions in which the instructions were mastery-approach focused but in a controlling style, or performance-approach focused with either a controlling or autonomy-supportive style. Study 2, which was set in a laboratory context with an audio working memory task and university students from a range of faculties, replicated this core finding. Up to now, research has shown that an autonomy-supportive style to induce either mastery or performance goals can be beneficial for a positive emotional experience, low tension, and better concentration on a task (expressed by fewer mistakes; Benita et al., 2014). It has also shown that inducing performance goals in an autonomy-supportive manner predict higher tendency to share information with playmates compared to when performance goals are induced in a controlling style (Sommet et al., 2019). An indication of the superiority of AM on task accuracy compared to CM and autonomy-support and CP condition. Our studies, however, extended this finding by, firstly, replicating in two different contexts (classroom and laboratory) that students’ dishonest behavior is lower when we promote mastery goals in an autonomy-supportive way than when we promote either CM goals or autonomy-supportive or CP goals.

These results underscore of considering both the goal that is promoted and the autonomy-supportive way of such a promotion, when we want to understand when achievement goals relate to task cheating. As such, they add to the burgeoning literature on Goal Complex (e.g., Vansteenkiste et al., 2010a; Michou et al., 2014; Delrue et al., 2016) by adding this experimental, goal-setting approach, and intimating that people’s behavior as well as their motivational responses to situations is a consequence of both goal content and goal reason that the induction style implies.

Secondly, they also extend the literature on the Goal Complex by showing that goal setting and the underlying quality of motivation can have an impact not only on self-reported learning strategies, emotional experience, or even cheating (Vansteenkiste et al., 2010b; Michou et al., 2014; Oz et al., 2016), but also on unethical behavior manifested during a specific task. On a practical level, they add to the literature on learning and teaching methodology as they provide the potential for concrete advice in training trainers to be autonomy supportive and learning oriented. This is highly relevant not just in the classroom, but also in sport coaching, the work environment, industrial, and research, in fact anywhere where people are receiving instructions to carry out tasks.

However, the fact that the effect only lasted for the first series of exercises alerts us to the short-lived nature of such influence, indicating peripheral processing (Petty and Cacioppo, 1986). This implies that for instructions to be efficient in reducing cheating, they need to be reinforced on a very regular basis during the task as well as before. The same goes for other such pre-task primes as the 10 commandments, as have been used by Mazar et al. (2008) and even honor code reminders (McCabe and Trevino, 1993).

### Values Matter

Our second finding in Study 1 was that another factor that needs to be taken into account is the degree of prior socialization learners have undergone. Notably, those who are already socialized to value competition, social image, wealth, and power may be less susceptible to task instruction manipulation. In effect, we found that the effect of task instructions was more powerful for participants who were relatively low in adherence to self-enhancement values (achievement, power, face) than for those who adhered more to these values. In addition, in the condition in which the least overall cheating was found, self-enhancement value adherence was a factor that positively predicted cheating, implying that the higher people are in their adhesion to this value relative to others, the less they are open to being influenced by the autonomy-related contextual cues coming from task instructions. Their internalized goal, to outperform others, may overpower the instructions and, especially when faced with task difficulties they are more likely to have recourse to unethical methods. This result reflects previous work relating adherence to self-enhancing values to cheating (Pulfrey and Butera, 2013) as well as research studies that show that competition is related to cheating (Perry et al., 1990). This moderation effect also provides an echo to the findings of Barron and Harackiewicz (2001), in which individual achievement orientation moderated the effect of goal assignation on performance. As such, it points to the potential usefulness of taking individual differences into consideration when attempting to assess the impact of interventions.

Our finding in Study 2 that self-enhancement values predict cheating in the second part of the exercises reinforces this argument. These results point to the ingrained nature of the self-enhancement value adherence-cheating relationship and, indeed, in this study we don’t even see interaction effects between self-enhancement value adherence and experimental condition, implying that here we see that previous socialization has inculcated unethical habits, which may not be so easily countered by a surface, contextual manipulation. The fact that participants high in self-enhancement value adherence even mis-remembered the goal orientation instructions for the task emphasizes how far this can go. In their role as life goals, values are deeply embedded in our psyche and relatively slow to change (Schwartz, 1992, 2006) and so attempts to bring about value change and behavior change that is contrary to these values are not a one-shot deal. As we already know from Petty and Cacioppo (1986) Elaboration Likelihood Model, for contextual primes to have a longer lasting influence and really create durable change, they either need to engage the individual in central processing that instigates a questioning and cognitive conflict, or else be reinforced constantly and in a situated, appropriate way in the social environment (Papies, 2016) with the aim of new habit formation.

A third point of interest that arises in these studies is the role of manipulation checks and participant recall of them. In Study 1 we see that participants did not recall the manipulation checks at the end of the entire task and, if anything, seemed likely to report their own goals. In Study 2, participants accurately remembered the manipulations immediately after reading them, which implies that a certain influence was exerted in the first part of the task, as can be seen in the results of Part 1. However, mid-way through the recall was already less accurate and by the end of the task, there was no recall. This alerts us to the very ephemeral nature of priming in task instructions and the need to find ways of ensuring that participants actually use a more central form of deep processing of task instructions, if we wish them to really take in the content of the instructions.

### Limitations

As always, there are a number of limitations to these studies. Firstly, due to financial constraints, the sample size of both studies is adequate to measure main effects, but with interaction effects, we may not be dealing with sufficient participants per condition in order to draw firm conclusions from our interaction results. However, the fact that we have a main effect of the autonomy-supportive/mastery contrast that replicates is a strong indicator that this core finding has value.

Secondly, the fact that the manipulation check did not work in Study 1 can be attributed to either a defensive reaction of participants not to admit, for example, that they wanted to outperform others to feel better about themselves (performance-approach goal for controlled reason) or to a less strong condition induction. If the second is true, the causality of the found relations is under question. However, as the results of Study 1 replicated by Study 2, where a more appropriate manipulation check was used, there are indications that indeed, an AM induction causes less cheating in the short term.

As this research was particularly interested in the impact of goal-related task instructions on cheating behavior, a third limitation is the fact that participants did not receive formal feedback concerning their performance between sets 1 and 2 of the exercises. However, in both tasks participants were able to gauge fairly accurately their raw score performance (for example, max six out of six puzzles in Set 1 Study 1), which meant they had some idea of how they had done. Providing appropriate formal feedback and reinforcing the goals set between Sets 1 and 2 of the exercises would be likely to produce more marked effects.

A fourth limitation, worth exploring, is the fact that we see less strong effects of the experimental manipulation in the laboratory study, and a stronger over-riding effect of demographic variables that are known to predict cheating, namely self-enhancement value adherence and gender. While this could be due to the task being different, it could also be due to the fact that we were more removed from the classroom setting, there was not a “teacher” present and the task was computerized. This calls for a continued effort to carry out studies not only in psychology laboratories but also in the classroom setting. The latter may be more messy and difficult to control, but it has the advantage of a strong ecological validity.

Finally, in this study, we have focused on the role of performance- and mastery-approach goal inductions, as these are likely to be more present in the classroom setting. Further research could explore the role of autonomy-supportive versus controlling performance- and mastery-avoidance goal setting in task instructions and their impact on cheating, as well as the way participants actually interpret the goals set in tasks.

## Conclusion

Cheating in education consumes time, money, and other resources, as well as constituting a serious threat to the legitimacy of academic qualifications and it is still on the rise with new technologies making it easier to get round rules (Marsh, 2017a). The rise of essay-mill sites in particular is posing a problem that needs addressing on a national scale and universities are having a hard time keeping up with cheaters’ ingenuity (Marsh, 2017b). We believe that a continued effort to understand more about the factors seducing students to cheat is crucial in combatting cheating effectively, as where there is a will, there’s always a way. The goal complex approach has provided a timely advance in motivation theory in general in enabling us to tease apart goal content promotion and goal style promotion, thus fine-tuning our understanding of the motivational environment that drives individuals (Elliot and Thrash, 2001; Vansteenkiste et al., 2014a). Likewise, in the specific case of cheating, we see that it may not be enough to draw on established wisdom and focus on mastery-approach goals, if we wish to reduce cheating a maximum. Pressure to improve, albeit for external reward or social approval, can still be an incitement to cheat and an equal focus on autonomy-support as well as mastery-approach goals seems a necessary condition to reduce cheating a maximum (see also Delrue et al., 2016).

Furthermore, although goals and motivational patterns can become ingrained over time, ending up as stable traits, they are, like individual values, the result in part of ongoing, daily socialization. As such, anyone who is giving instructions regularly, teachers, trainers, managers, or doctors, for example, should be vigilant as to what hidden messages may be coming across in the way they present tasks to those for whom they are responsible. The concept of nudging, that is to say, creating an architecture of choice to modify people’s *voluntary* behavior in ways that can be predicted (Thaler and Sunstein, 2009), is not only applicable in marketing, but can fruitfully be applied in learning contexts as another weapon in the war on cheating.

## Data Availability

The datasets generated for this study are available on request to the corresponding author.

## Ethics Statement

Regarding Experiment 1, an ethics approval was not required at the time the study was conducted as per applicable institutional and regional/national guidelines. The material for Experiment 1 was submitted for approval to the first author’s Head of Department, as well as to the teacher whose classes participated in the experiment. Both approved it. The second experiment and the overall project, including the manuscript write-up of Study 1, was officially approved by the Ethics Committee of the first author’s faculty at that time, the Faculty of Business and Economics of the University of Lausanne. Oral informed consent was obtained from all participants of the first study and written informed consent was obtained from all participants of the second study.

## Author Contributions

CP developed the literature review, created and carried out the two experimental studies, did the data analysis (Study 2), and wrote the manuscript. MV had the idea of exploring goal reasons and cheating, brought CP and AM together, helped conceptualize the first study, and critiqued and improved the manuscript. AM helped conceptualize the first study, worked on the data analysis of the first study, critiqued, revised, and improved the manuscript.

## Conflict of Interest Statement

The authors declare that the research was conducted in the absence of any commercial or financial relationships that could be construed as a potential conflict of interest.

## Footnotes

1 The *DEFF* for set 2 of the exercises is 3.09, which reflects the significant difference between classes 3 and 5, noted in the section “Results.” However, in set 2 alone, no significant effects of the experimental conditions emerge and they are only used as part of the aggregate score of total cheating.

2 All non-copyrighted experimental materials as well as data files will be made available on request, without any undue reservation, to any qualified and interested researchers. As an official ethics approval was not required (no ethics committee) in the institution where the first author was working at the time Experiment 1 was carried out, the material for Experiment 1 was submitted for approval to the first author’s Head of Department, as well as to the teacher whose classes participated in the experiment. Both approved it. The second experiment and the overall project, including the manuscript write-up of Study 1, was officially approved by the Ethics Committee of the first author’s faculty at that time, the Faculty of Business and Economics of the University of Lausanne.

## Appendix

**TABLE:** The experimental manipulation of mastery-approach versus performance-approach goal content combined with controlling versus autonomy-supportive style in Study 1.

	**Performance-approach goals**	**Intrapersonal mastery-approach goals**
Autonomy-supportive style	**Over the page you will find a set of Spatial Exercises, *which most students find an interesting challenge*. There are two series of six spatial problems for you to try to solve individually. You *will be given* 8 min to solve each set of problems.** Success and achievement are all about who does best **and so and *you have the opportunity* to work individually on the puzzles, trying to** perform better than the other students.***Therefore, you can* look upon this task as a *personal challenge* and *see if you can*** get more puzzles correct than the others**. Focus on *the challenge of*** being among the top performers. **You will all receive the feedback scores once the whole study is completed.** Don’t forget that: **Success and achievement are all about who does best** and so *it’s a good idea to keep focusing on the challenge* of **being among the top performers.** You will receive all the feedback scores once the whole study is completed. You will have 8 min to complete the second set of six exercises.	**Over the page you will find a set of Spatial Exercises, *which most students find an interesting challenge.* There are two series of six spatial problems for you to try to solve individually. You *will be given* 8 min to solve each set of problems.** Success and achievement are all about personal improvement **and so *you have the opportunity* to work individually on the puzzles, trying to** improve your personal performance. **Therefore, *you can* look upon this task as a *personal challenge*, and *see if you can* improve your score by solving more puzzles in the second set than in the first. Focus on *the challenge of*** improving your solving skills in the second set**. You will all receive the feedback scores once the whole study is completed.** Don’t forget that: **Success and achievement are all about personal improvement** and so *it’s a good idea to keep focusing on the challenge* of **improving your solving skills in the second set.** You will receive all the feedback scores once the whole study is completed. You will have 8 min to complete the second set of six exercises.
Controlling style	**Over the page you will find a Spatial Exercise *Test*, which *evaluates* your capacity for logical spatial insight. There are two series of six spatial problems you *must* try to solve individually. You *will have to* finish each set of problems within 8 min.** Success and achievement are all about who does best **and so *you are expected* to work individually on the puzzles, and *to prove* that you can** perform better than the other students.** Therefore, *you ought to* look upon this task as a way of *impressing others* by** getting more puzzles correct than the others.** Focus on the fact that *you need to* be** among the top performers.** You will all receive the feedback scores once the whole study is completed.** Don’t forget that: **Success and achievement are all about who does best** and so *you need to* keep focusing on **being among the top performers**. You will receive all the feedback scores once the whole study is completed. You will have 8 min to complete the second set of six exercises.	**Over the page you will find a Spatial Exercise *Test*, which *evaluates* your capacity for logical spatial insight. There are two series of six spatial problems you *must* try to solve individually. You *will have to* finish each set of problems within 8 min.** Success and achievement are all about personal improvement **and so *you are expected to* work individually on the puzzles, and *to prove* that you can** improve on your personal performance. **Therefore, *you ought to* look upon this task as a way of *impressing others* by** solving more puzzles in the second set than in the first. **Focus on the fact that *you need to*** improve your solving skills in the second set. You will all receive the feedback scores once the whole study is completed. Don’t forget that: **Success and achievement are all about personal improvement** and *so you need to* keep focusing **on improving your solving skills in the second set.** You will receive all the feedback scores once the whole study is completed. You will have 8 min to complete the second set of six exercises.

*Text in bold represents phrases relevant to the goal inductions. Text in italics represents phrases relevant to controlling versus autonomy-supportive style.*

## References

[B1] Anderman E. M. Danner F. (2008). Achievement goals and academic cheating. *Int. Rev. Soc. Psychol.* 21 155–180.

[B2] Anderman E. M. Griessinger T. Westerfield G. (1998). Motivation and cheating during early adolescence. *J. Educ. Psychol.* 90 84–93. 10.1016/j.ypmed.2016.12.042 28043828

[B3] Anderman E. M. Midgley C. (2004). Changes in self-reported academic cheating across the transition from middle school to high school. *Contemp. Educ. Psychol.* 29 499–517. 10.1016/j.cedpsych.2004.02.002 9237829

[B4] Barron K. E. Harackiewicz J. M. (2001). Achievement goals and optimal motivation: testing multiple goal models. *J. Person. Soc. Psychol.* 80:706. 10.1037//0022-3514.80.5.706 11374744

[B5] Benita M. Roth G. Deci E. L. (2014). When are mastery goals more adaptive? it depends on experiences of autonomy support and autonomy. *J. Educ. Psychol.* 106:258 10.1037/a0034007

[B6] Cameron A. C. Trivedi P. K. (2009). *Microeconometrics Using Stata.* College Station, TE: Stata Press.

[B7] Cowan N. (2010). The magical mystery four: how is working memory capacity limited, and why? *Curr. Direct. Psychol. Sci.* 19 51–57. 10.1177/0963721409359277 20445769 PMC2864034

[B8] Darnon C. Dompnier B. Delmas F. Pulfrey C. Butera F. (2009). Achievement goal promotion at university: social desirability and social utility of mastery and performance goals. *J. Person. Soc. Psychol.* 96:119. 10.1037/a0012824 19210069

[B9] Davy J. A. Kincaid J. F. Smith K. J. Trawick M. A. (2007). An examination of the role of attitudinal characteristics and motivation on the cheating behavior of business students. *Ethics Behav.* 17 281–302. 10.1080/10508420701519304

[B10] DeAndrea D. C. Carpenter C. Shulman H. Levine T. R. (2009). The relationship between cheating behavior and sensation-seeking. *Person. Individ. Differ.* 47 944–947. 10.1016/j.paid.2009.07.021

[B11] Delrue J. Mouratidis A. Haerens L. De Muynck G.-J. Aelterman N. Vansteenkiste M. (2016). Intrapersonal achievement goals and underlying reasons among long distance runners: their relation with race experience, self-talk, and running time. *Psychologica Belgica* 56 288–310. 10.5334/pb.280 30479441 PMC5853908

[B12] Dompnier B. Darnon C. Butera F. (2009). Faking the desire to learn: a clarification of the link between mastery goals and academic achievement. *Psychol. Sci.* 20 939–943. 10.1111/j.1467-9280.2009.02384.x 19538435

[B13] Dweck C. S. (1999). *Self-theories Their Role in Motivation, Personality, and Development.* Philadelphia, PA: Psychology Press.

[B14] Elliot A. J. (2005). “A conceptual history of the achievement goal construct,” in *Handbook of Competence and Motivation*, eds Elliot A. J. Dweck C. S. (New York, NY: Guilford Press), 52–72.

[B15] Elliot A. J. Harackiewicz J. M. (1994). Goal setting, achievement orientation, and intrinsic motivation: a mediational analysis. *J. Person. Soc. Psychol.* 66 968–980. 10.1037/0022-3514.66.5.968 8014838

[B16] Elliot A. J. McGregor H. A. (2001). A 2 × 2 achievement goal framework. *J. Person. Soc. Psychol.* 80 501–519. 10.1037/0022-3514.80.3.501 11300582

[B17] Elliot A. J. Murayama K. Pekrun R. (2011). A 3 × 2 achievement goal model. *J. Educ. Psychol.* 103:632.

[B18] Elliot A. J. Thrash T. M. (2001). Achievement goals and the hierarchical model of achievement motivation. *Educ. Psychol. Rev.* 13 139–156.

[B19] Gaudreau P. (2012). Goal self-concordance moderates the relationship between achievement goals and indicators of academic adjustment. *Learn. Individ. Differ.* 22 827–832. 10.1016/j.lindif.2012.06.006

[B20] Gillet N. Lafreniere M.-A. K. Vallerand R. J. Huart I. Fouquereau E. (2012). The effects of autonomous and controlled regulation of performance-approach goals on well-being: a process model. *Br. J. Soc. Psychol.* 51 1–21. 10.1111/bjso.12018 23121496

[B21] Grolnick W. S. Ryan R. M. (1989). Parent styles associated with children’s self-regulation and competence in school. *J. Educ. Psychol.* 81 143–154. 10.1037/fam0000524 30869916

[B22] Hulleman C. S. Schrager S. M. Bodmann S. M. Harackiewicz J. M. (2010). A meta-analytic review of achievement goal measures: different labels for the same constructs or different constructs with similar labels? *Psychol. Bull.* 136:422. 10.1037/a0018947 20438145

[B23] Jordan A. E. (2001). College student cheating: the role of motivation, perceived norms, attitudes, and knowledge of institutional policy. *Ethics Behav.* 11 233–247. 10.1207/s15327019eb1103_3

[B24] Kaplan A. Middleton M. J. Urdan T. Midgley C. (2002). Achievement goals and goal structures. *Goals Goal Struct. Patter. Adap. Learn.* 17 21–53.

[B25] Kasser T. Ryan R. M. Zax M. Sameroff A. J. (1995). The relations of maternal and social environments to late adolescents’ materialistic and prosocial values. *Dev. Psychol.* 31 907–914. 10.1037//0012-1649.31.6.907

[B26] Klein H. A. Levenburg N. M. McKendall M. Mothersell W. (2007). Cheating during the college years: how do business school students compare? *J. Business Ethics* 72 197–206. 10.1007/s10551-006-9165-7

[B27] Lobel T. E. Levanon I. (1988). Self-esteem, need for approval, and cheating behavior in children. *J. Educ. Psychol.* 80 122–123. 10.1037/0022-0663.80.1.122

[B28] Mandard S. (2013). *Lance Armstrong: “Le Tour de France? Impossible de gagner sans dopage”. Le Monde.* Available at: https://www.lemonde.fr/sport/article/2013/06/28/lance-armstrong-le-tour-de-france-impossible-de-gagner-sans-dopage_3438046_3242.html (accessed June 28, 2013).

[B29] Marsh S. (2017a). *More university Students are Using Tech to Cheat in Exams The Guardian.* Available at: https://www.theguardian.com/education/2017/apr/10/more-university-students-are-using-tech-to-in-exams (accessed April 10, 2017).

[B30] Marsh S. (2017b). *Universities Urged to Block Essay-Mill Sites in Plagiarism Crackdown.* Available at: https://www.theguardian.com/education/2017/oct/09/universities-urged-to-block-essay-mill-sites-in-plagiarism-crackdown (accessed October 9, 2017).

[B31] Mazar N. Amir O. Ariely D. (2008). The dishonesty of honest people: a theory of self-concept maintenance. *J. Mark. Res.* 45 633–644. 10.1509/jmkr.45.6.633

[B32] McCabe D. L. Trevino L. K. (1993). Academic dishonesty: honor codes and other contextual influences. *J. Higher Educ.* 64 522–538. 10.1080/00221546.1993.11778446

[B33] Michou A. Vansteenkiste M. Mouratidis A. Lens W. (2014). Enriching the hierarchical model of achievement motivation: autonomous and controlling reasons underlying achievement goals. *Br. J. Educ. Psychol.* 84 650–666. 10.1111/bjep.12055 25251866

[B34] Murayama K. Elliot A. J. (2009). The joint influence of personal achievement goals and classroom goal structures on achievement-relevant outcomes. *J. Educ. Psychol.* 101 432–447. 10.1037/a0014221

[B35] Murdock T. Miller A. Goetzinger A. (2007). Effects of classroom context on university students’ judgments about cheating: mediating and moderating processes. *Soc. Psychol. Educ.* 10 141–169. 10.1007/s11218-007-9015-1

[B36] Murdock T. B. Hale N. M. Weber M. J. (2001). Predictors of cheating among early adolescents: academic and social motivations. *Contemp. Educ. Psychol.* 26 96–115. 10.1006/ceps.2000.1046 11161642

[B37] Murdock T. B. Miller A. Kohlhardt J. (2004). Effects of classroom context variables on high school students’ judgments of the scceptability and likelihood of cheating. *J. Educ. Psychol.* 96 765–777. 10.1037/0022-0663.96.4.765

[B38] Niiya Y. Ballantyne R. North M. S. Crocker J. (2008). Gender, contingencies of self-worth, and achievement goals as predictors of academic cheating in a controlled laboratory setting. *Basic Appl. Soc. Psychol.* 30 76–83. 10.1080/01973530701866656

[B39] Orosz G. Farkas D. Roland-Lévy C. (2013). Are competition and extrinsic motivation reliable predictors of academic cheating? *Front. Psychol.* 4:87. 10.3389/fpsyg.2013.00087 23450676 PMC3583185

[B40] Oz A. O. Lane J. F. Michou A. (2016). Autonomous and controlling reasons underlying achievement goals during task engagement: their relation to intrinsic motivation and cheating. *Educ. Psychol.* 36 1160–1172. 10.1080/01443410.2015.1109064

[B41] Papies E. K. (2016). Goal priming as a situated intervention tool. *Curr. Opin. Psychol.* 12 12–16. 10.1016/j.copsyc.2016.04.008 27144729 PMC5214881

[B42] Peled Y. Eshet Y. Barczyk C. Grinautski K. (2019). Predictors of Academic Dishonesty among undergraduate students in online and face-to-face courses. *Comput. Educ.* 131 49–59. 10.1016/j.compedu.2018.05.012

[B43] Perry A. R. Kane K. M. Bernesser K. J. Spicker P. T. (1990). Type a behavior, competitive achievement-striving, and cheating among college students. *Psychol. Rep.* 66 459–465. 10.2466/pr0.1990.66.2.459 2349335

[B44] Petty R. E. Cacioppo J. T. (1986). *The Elaboration Likelihood Model of Persuasion in Communication anPerd Persuasion.* New York, NY: Springer, 1–24.

[B45] Pope D. C. (2000). *Doing School: “Successful” Students’ Experiences of the High School Curriculum.* Doctoral dissertation, Stanford, CA: Stanford University.

[B46] Pulfrey C. Butera F. (2013). Why neo-liberal values lead to cheating: a motivational account. *Psychol. Sci.* 24 2153–2162. 10.1177/0956797613487221 24058068

[B47] Pulfrey C. Butera F. (2016). When and why people don’t accept cheating: self-transcendence values, social responsibility, mastery goals and attitudes towards cheating. *Motivat. Emot.* 40 438–454. 10.1007/s11031-015-9530-x

[B48] Pulfrey C. Darnon C. Butera F. (2013). Autonomy and task performance: explaining the impact of grades on intrinsic motivation. *J. Educ. Psychol.* 105 39–57. 10.1037/a0029376

[B49] Pulfrey C. Durussel K. Butera F. (2018). The good cheat: benevolence and the justification of collective cheating. *J. Educ. Psychol.* 110 764–784. 10.1037/edu0000247

[B50] Ryan R. M. Connell J. P. (1989). Perceived locus of causality and internalization: examining reasons for acting in two domains. *J. Person. Soc. Psychol.* 57 749–761. 10.1037//0022-3514.57.5.749 2810024

[B51] Ryan R. M. Deci E. L. (2000). Intrinsic and extrinsic motivations: classic definitions and new directions. *Contemp. Educ. Psychol.* 25 54–67. 10.1006/ceps.1999.1020 10620381

[B52] Ryan R. M. Deci E. L. (2017). *Self-Determination Theory: Basic Psychological Needs in Motivation, Development, and Wellness.* New York, NY: Guilford Publications.

[B53] Savard A. Joussemet M. Pelletier J. E. Mageau G. A. (2013). The benefits of autonomy support for adolescents with severe emotional and behavioral problems. *Motivat. Emot.* 37 688–700. 10.1007/s11031-013-9351-8

[B54] Schrank F. A. Wendling B. J. (2018). “The Woodcock–Johnson IV: tests of cognitive abilities, tests of oral language, tests of achievement,” in *Contemporary Intellectual Assessment: Theories, Tests, and Issues*, eds Flanagan D. P. McDonough E. M. (New York, NY: The Guilford Press).

[B55] Schraw G. Wadkins T. Olafson L. (2007). Doing the things we do: a grounded theory of academic procrastination. *J. Educ. Psychol.* 99:12 10.1037/0022-0663.99.1.12

[B56] Schwartz S. H. (1992). Universals in the content and structure of values: theoretical advances and empirical tests in 20 countries. *Adv. Exp. Soc. Psychol.* 25 1–65. 10.1016/s0065-2601(08)60281-6 17874285

[B57] Schwartz S. H. (2006). Les valeurs de base de la personne: théorie, mesures et applications. *Revue française de Sociologie* 47 929–968.

[B58] Schwartz S. H. Cieciuch J. Vecchione M. Davidov E. Fischer R. Beierlein C. (2012). Refining the theory of basic individual values. *J. Person. Soc. Psychol.* 103 663–688. 10.1037/a0029393 22823292

[B59] Senko C. (2016). “Achievement goal theory: a story of early promises, eventual discords, and future possibilities,” in *Handbook of Motivation at School*, Vol. 2 eds Wentzel K. R. Miele D. B. (New York, NY: Taylor & Francis), 75–95.

[B60] Senko C. Dawson B. (2017). Performance-approach goal effects depend on how they are defined: meta-analytic evidence from multiple educational outcomes. *J. Educ. Psychol.* 109 574 10.1037/edu0000160

[B61] Shanks D. R. Newell B. R. Lee E. H. Balakrishnan D. Ekelund L. Cenac Z. (2013). Priming intelligent behavior: an elusive phenomenon. *PLoS One* 8:e56515. 10.1371/journal.pone.0056515 23637732 PMC3634790

[B62] Sommet N. Elliot A. J. (2017). Achievement goals, reasons for goal pursuit, and achievement goal complexes as predictors of beneficial outcomes: is the influence of goals reducible to reasons? *J. Educ. Psychol.* 109 1141–1162. 10.1037/edu0000199

[B63] Sommet N. Nguyen D. Fahrni K. Jobin M. Nguyen H.-P. Sehaqui H. (2019). When and why performance goals predict exploitation behaviors: an achievement goal complex analysis of the selection function of assessment. *Motivat. Emot.* 43 266–284. 10.1007/s11031-018-9742-y

[B64] Spray C. M. John Wang C. K. Biddle S. J. Chatzisarantis N. L. (2006). Understanding motivation in sport: an experimental test of achievement goal and self determination theories. *Eur. J. Sport Sci.* 6 43–51. 10.1080/17461390500422879

[B65] Thaler R. H. Sunstein C. R. (2009). *Nudge Improving Decisions about Health, Wealth, and Happiness.* London: Penguin Group.

[B66] Urdan T. C. (1997). Examining the relations among early adolescent students’ goals and friends’ orientation toward effort and achievement in school. *Contemp. Educ. Psychol.* 22 165–191. 10.1006/ceps.1997.0930

[B67] Van Yperen N. W. (2006). A novel approach to assessing achievement goals in the context of the 2 × 2 framework: identifying distinct profiles of individuals with different dominant achievement goals. *Person. Soc. Psychol. Bull.* 32 1432–1445. 10.1177/0146167206292093 17030886

[B68] Van Yperen N. W. Blaga M. Postmes T. (2015). A meta-analysis of the impact of situationally induced achievement goals on task performance. *Hum. Perform.* 28 165–182. 10.1080/08959285.2015.1006772

[B69] Van Yperen N. W. Elliot A. J. Anseel F. (2009). The influence of mastery-avoidance goals on performance improvement. *Eur. J. Soc. Psychol.* 39 932–943. 10.1002/ejsp.590

[B70] Van Yperen N. W. Hamstra M. R. van der Klauw M. (2011). To win, or not to lose, at any cost: the impact of achievement goals on cheating. *Br. J. Manag.* 22 S5–S15.

[B71] Vansteenkiste M. Lens W. Elliot A. J. Soenens B. Mouratidis A. (2014a). Moving the achievement goal approach one step forward: toward a systematic examination of the autonomous and controlled reasons underlying achievement goals. *Educ. Psychol.* 49 153–174. 10.1080/00461520.2014.928598

[B72] Vansteenkiste M. Mouratidis A. Van Riet T. Lens W. (2014b). Examining Correlates of Game-to-Game Variation in Volleyball Players’ Achievement Goal Pursuit and Underlying Autonomous and Controlling Reasons. *J. Sport Exerc. Psychol.* 36 131–145. 10.1123/jsep.2012-0271 24686950

[B73] Vansteenkiste M. Mouratidis A. Lens W. (2010a). Detaching reasons from aims: fair play and well-being in soccer as a function of pursuing performance-approach goals for autonomous or controlling reasons. *J. Sport Exerc. Psychol.* 32 217–242. 10.1123/jsep.32.2.217 20479479

[B74] Vansteenkiste M. Smeets S. Soenens B. Lens W. Matos L. Deci E. L. (2010b). Autonomous and controlled regulation of performance-approach goals: their relations to perfectionism and educational outcomes. *Motivat. Emot.* 34 333–353. 10.1007/s11031-010-9188-3

[B75] Vansteenkiste M. Sierens E. Soenens B. Luyckx K. Lens W. (2009). Motivational profiles from a self-determination perspective: the quality of motivation matters. *J. Educ. Psychol.* 101 671–688. 10.1037/a0015083

[B76] Vansteenkiste M. Zhou M. Lens W. Soenens B. (2005). Experiences of autonomy and control among Chinese learners: vitalizing or immobilizing? *J. Educ. Psychol.* 97:468 10.1037/0022-0663.97.3.468

[B77] Whitley B. E. (1998). Factors associated with cheating among college students: a review. *Res. Higher Educ.* 39 235–274.

[B78] Whitley B. E. Nelson A. B. Jones C. J. (1999). Gender differences in cheating attitudes and classroom cheating behavior: a meta-analysis. *Sex Roles* 41 657–680.

